# Controlled Structure and Growth Mechanism behind Hydrothermal Growth of TiO_2_ Nanorods

**DOI:** 10.1038/s41598-020-64510-6

**Published:** 2020-05-15

**Authors:** Aschariya Prathan, Jongrak Sanglao, Tao Wang, Chawalit Bhoomanee, Pipat Ruankham, Atcharawon Gardchareon, Duangmanee Wongratanaphisan

**Affiliations:** 1 Department of Physics and Materials Science, Faculty of Science, Chiang Mai University, Chiang Mai, 50200 Thailand; 2 Ph.D. Program in Physics, Department of Physics and Materials Science, Chiang Mai University, Chiang Mai, 50200 Thailand; 3 School of Materials Science and Engineering, Wuhan University of Technology, Wuhan, 430070 China; 4 Visiting Professor at Department of Physics and Materials Science, Faculty of Science, Chiang Mai University, Chiang Mai, 50200 Thailand; 5 Thailand Center of Excellence in Physics (ThEP center), Ministry of Higher Education, Science, Research and Innovation, Bangkok, 10400 Thailand

**Keywords:** Materials science, Physics

## Abstract

Fabrication of uniform vertically-aligned titanium dioxide nanorods (TiO_2_ NRs) was achieved by hydrothermal growth on a fluorine-doped tin oxide (FTO) glass substrate. The substrate was coated by a TiO_2_ seed layer composed of titanium (IV) butoxide (TBO) as a precursor in an HCl solution. To reduce the amount of toxic substances used in this work, a minimal amount of HCl was used. On a larger scale, this method would require less precursor and therefore be a cost-savings. The aim of the present work is to achieve high crystalline orientations of TiO_2_ NRs for low quantities of both TBO precursor and HCl solutions. Results showed that the 0.7% TBO TiO_2_ NRs after 1.5 h of hydrothermal treatment exhibited the optimal crystalline orientation along [001] while the (002) plane is the dominant facet. The results demonstrate high transmittance of visible light and well-formed crystalline structures that offer a fast electron pathway along the length of the TiO_2_ NRs with less grain boundaries. Lastly, TiO_2_ NRs and their growth mechanism are discussed. This work offers a promising hydrothermal method for growing well-aligned TiO_2_ single-crystal NRs that can be employed in solar cell applications.

## Introduction

There is a growing demand for new materials to use in applications that meet today’s energy and environmental challenges. In particular, wide band gap semiconductors, using very promising materials, are demonstrating impressive properties for example, high electron mobility, large band gaps and reasonably good conductivity. Among semiconducting wide band-gap materials, titanium dioxide (TiO_2_) and zinc oxide (ZnO) are alternative materials used as electron transporting layers with suitable energy levels relative to Perovskite solar cells (PSC). Due to the superior electron transfer capability of each material, which is a crucial factor for PSC performance, many approaches have been found allowing TiO_2_ and ZnO to be used together to enhance the overall power conversion efficiency (PCE) of PSCs. Even though ZnO layers exhibit higher electron mobility compared to TiO_2_, ZnO-based PSCs exhibit poor stability, which is a serious problem. If a Perovskite layer is created from a composition of methyl ammonium $({{\rm{CH}}}_{3}{{\rm{NH}}}_{3}^{+})$ and lead triiodide $({{\rm{PbI}}}_{3}^{-})$ deposited on a ZnO layer, it can easily decompose during thermal treatment. However, there is less of this thermal decomposition for Perovskite and TiO_2_ interfaces. TiO_2_ has an acidic surface while a ZnO surface shows basic properties with high adsorption of positive charges^1^. When Perovskite is exposed on a ZnO layer, a deprotonation reaction with ${{\rm{CH}}}_{3}{{\rm{NH}}}_{3}^{+}$ occurs which could break the ionic interaction between ${{\rm{CH}}}_{3}{{\rm{NH}}}_{3}^{+}$ and ${{\rm{PbI}}}_{3}^{-}$ and as a result, it can obstruct the crystal formation of the Perovskite^2^. TiO_2_ becomes the better choice and overwhelmingly interesting for its fundamental aspects and applications as well as the main motivation of this work. Moreover, TiO_2_ is an ideal candidate for the large-scale manufacture of chemicals due to its stability, non-toxicity and the efficacy of its photoactivity^2,3^. Recent achievements in solar cell research have focused on the use of TiO_2_. However, the functionality for native ultraviolet (UV) light (λ < 387 nm) can excite TiO_2_ forms. Due to the wide band gap of TiO_2_, UV light is less than 5% of solar irradiance. The visible light and near infrared photons cannot be absorbed by TiO_2_ forms but UV or higher-energy can^2^.

**Figure 1:**
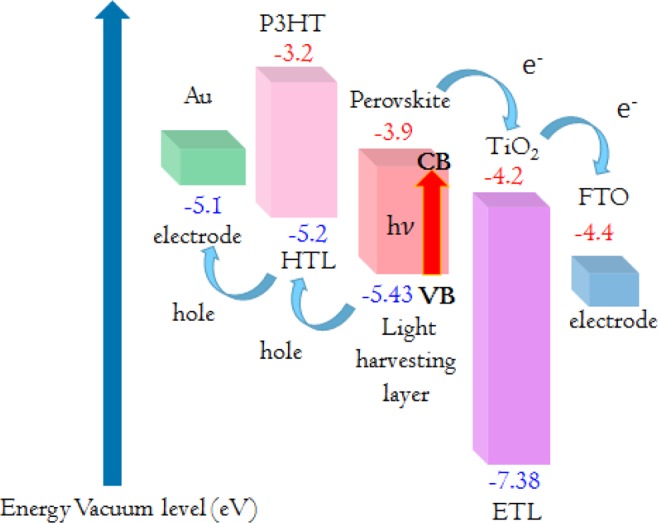
Band diagram of conventional Perovskite solar cell.

TiO_2_ can serve as a medium in highly efficient PSCs. The Perovskite structure is defined as an ABX_3_ compound consisting of a corner-sharing BX_6_ octahedra, where X is an anion or halogen (generally iodine) with A and B cations of different sizes (A being larger than B). The larger A cation is usually an organic methylammonium ${{\rm{CH}}}_{3}{{\rm{NH}}}_{3}^{+}$ ion. Pb^2+^ has been used extensively as a B cation because it offers durable and effective protection against oxidation. For CH_3_NH_3_PbI_3_ Perovskite, the cubic phase forms only at high temperatures (>330 K) producing a band gap energy of 1.55 eV with efficient absorption in the visible range^4,5^. Perovskites are placed between electron transporting layers (ETLs), which are usually composed of TiO_2_, and hole transporting layers (HTLs) that generally use Poly(3-hexylthiophene) (P3HT). Perovskite is an absorber layer that converts light energy to produce electron-hole pairs. Then, electrons and holes are transferred to ETLs and HTLs, respectively. Because the conduction band of TiO_2_ is lower than the conduction band of Perovskite, electrons are transferred from the Perovskite layer to the ETL and finally collected by the FTO anode^6,7^. Holes are transferred through the metal electrode due to the fact that the valence band of P3HT is higher than the valence band of Perovskite as shown in Fig. 1. Finally, the solar cell can supply power to an external system. For high performance solar cells, effective ETLs should have good electron mobility and energy levels for ETLs and Perovskite materials that are well matched to improve the electron transfer and block the holes. Last but not least, ETLs should have high transparency in visible light regions which reduces the absorption of visible light before it travels to the Perovskite active layer^7^.

Native thin TiO_2_ layers that have formed in a mesoporous arrangement are the basis for constructing traditional ETLs. Mesoporous layers (MSLs) contain many grain boundaries formed from the large numbers of small-sized nanoparticles and it is these boundaries within the layers that prevent a direct contact between the metal substrate and the Perovskite^8^. An obstructed flow causes electrons to be trapped at the grain boundary and degrades the charge transfer. It has been observed that TiO_2_ nanorods appear to have better characteristics than traditional TiO_2_ nanoparticles (NPs). For example, the fact that TiO_2_ NRs have less grain boundaries and longer lifetime electrons contributes to a higher quantity of charge carriers and consequently leads to an increased photo conversion efficiency^9–12^. Among one-dimensional (1D) TiO_2_ nanostructures, TiO_2_ NRs exhibit excellent electron transfer properties including high photoelectrochemical (PEC) performance and high electron lifetime^13^. Hydrothermally grown TiO_2_ NRs exhibit PEC performance almost six times higher than that of hydrothermally grown TiO_2_ nanotubes (NTs). Owing to the fact that the electron lifetime of TiO_2_ NRs was higher than that of TiO_2_ NTs about 10^4^ times and the rutile TiO_2_ of the NRs possessed lower intrinsic resistance^14^. Moreover, nanowires (NWs) generally have diameters in the order of tens of nanometers with aspect ratios of about 1000 while NRs have diameters in the range of 1–100 nm and the proportional relationship between width and height is about 5–100. Morphology control of NWs is difficult because of their extremely high aspect ratio and large inter-wire spacing. The significantly small and long architectures of NWs do not have a well-defined morphology causing NWs to collapse and hit each other. Previous studies have revealed that NWs obtained from a hydrothermal approach appeared as long fibers laying lengthwise and overlapping each other in a horizontal direction parallel to the substrate surface^14,15^. These irregular orientations may promote random walk and provide more scattering sites for electrons in the nanowire structures thus causing a high probability of charge recombination. Recently, it has been reported that high performance PSCs showing an 18.69% efficiency had used single-crystalline TiO_2_ NRs as the ETL which supports the idea that TiO_2_ NR architectures are dominating and interesting choices^16^. Therefore, NRs are more interesting because their size, shape and crystallographic orientation can be more easily tuned to vertical orientations. As a result, nanorods can serve as good ETLs that enable orderly electron transfer. Then, the well-aligned orientations of NRs, with optimized sizes and well-spaced nanorods, could promote charge carrier mobility in PSCs.

Therefore, this work aims to achieve a highly crystalline structure within TiO_2_ NRs and a crystallographic orientation which could promote high transmittance in TiO_2_ film using a hydrothermal method^17^. Precursor concentration and growth time were varied to control the dimensions of the NRs. The nanorod architectures were optimized to investigate the optical properties best suited to PSCs. The hydrothermal method is a good alternative to synthesis of NRs because this procedure is preferred over other techniques^15,18^. Single- and poly-crystallines can be produced in an autoclave at high pressure and a low temperature (<200 °C). This process eliminates the sintering process at a high temperature and is suitable for low cost manufacturing because the methodologies are simple and the equipment needs are reduced^15,18^. In order to reduce the quantity of harmful chemicals used, the synthesis will be conducted with a minimal amount of hydrochloric acid (HCl) with TiO_2_ seed layers designed to be the supporting base. Several factors affect the development of TiO_2_ NRs such as precursor concentrations, calcination and reaction times and these will be optimized to control the growth of the TiO_2_ NRs. Moreover, the growth mechanism of TiO_2_ NRs under each condition will be used to explain the phenomena that occur during the hydrothermal reaction. The prepared TiO_2_ NRs that exhibit very dominant structural and optical properties would therefore be the ones that are suitable for use as ETLs in PSCs.

**Figure 2:**
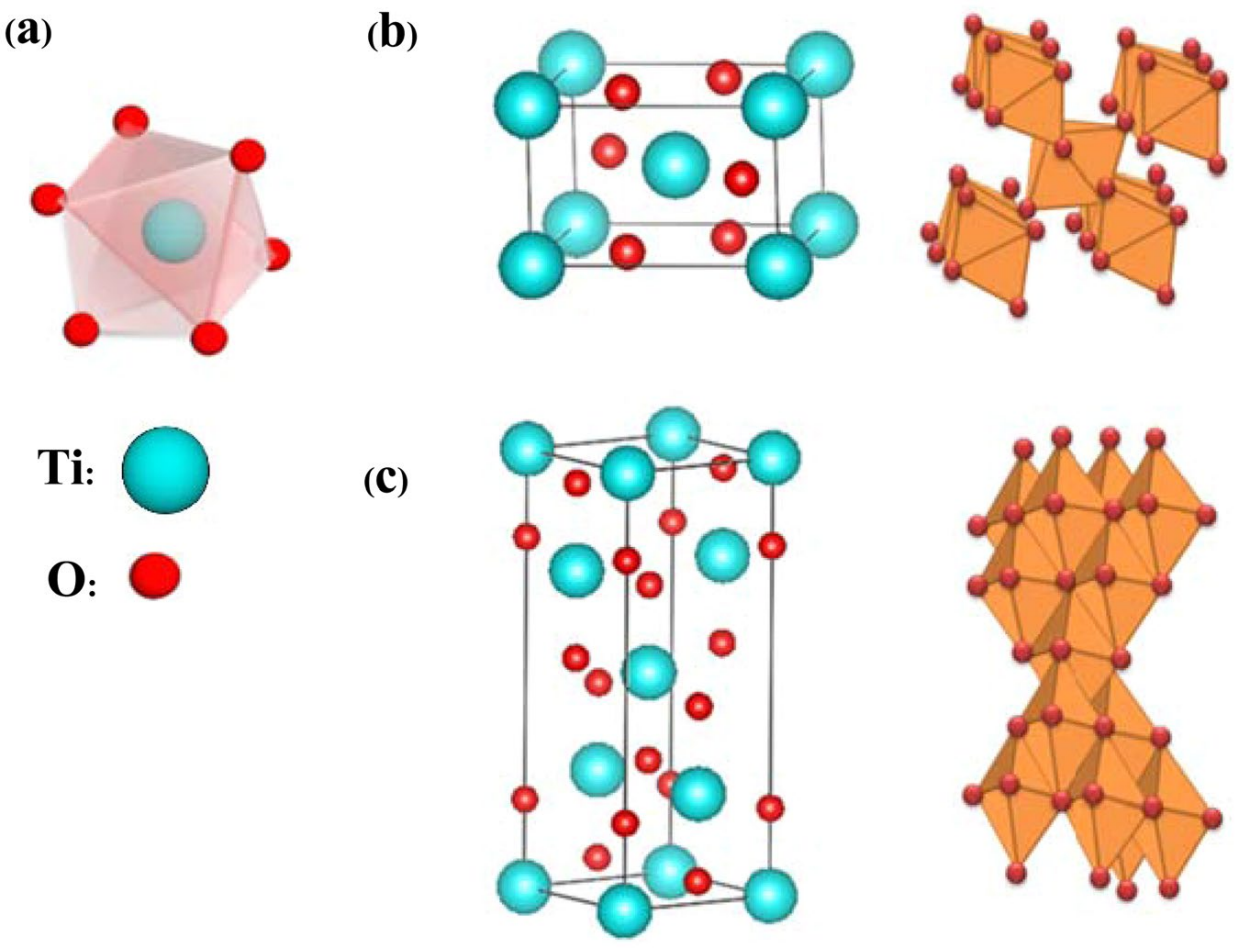
Molecular structures of TiO_2_ (**a**) octahedral building block that consists of one Ti atom surrounded by six O atoms (**b**) rutile and (**c**) anatase.

The physical properties of TiO_2_ are significantly dependent on the crystal structure. Two phases dominate for TiO_2_ at ambient conditions are known as rutile and anatase. Chains of TiO_6_ octahedra are the building blocks that make up their tetragonal lattice structures. One Ti^4+^ ion is surrounded by an octahedron of six O^2-^ ions. In the rutile phase, two opposite edges of each octahedron link at a corner of an oxygen atom, forming linear chains of octahedra at each corner as shown in Fig. 2(b). On the other hand, anatase has no corner sharing but shows four edges shared per octahedron (faces sharing). The anatase structure consists of zigzag chains of TiO_6_ octahedra that link to each other with edge-shared bonding. In this structure, there are larger internal spaces between the octahedra because there are more edges shared. As a result, its density packing is lower than that of rutile as shown in Fig. 2^19–21^. For nanorod structures, rutile exhibits an indirect band gap of 2.95 eV and anatase has a direct band gap of 3.5 eV^17^.

Generally, formation of TiO_2_ NRs using a hydrothermal procedure is carried out in hydrochloric acid (HCl) diluted with deionized water (DI) while Ti (IV) butoxide (TBO) with structural formula Ti(OCH_2_CH_2_CH_2_CH_3_)_4_ (or [Ti(RO)_4_]) is applied as a reactant. There are a number of approaches explaining growth mechanisms of TiO_2_, which are interesting and beneficial to this research. As the four-fold Ti precursor ([Ti(RO)_4_]) reacts with water, the coordination number of Ti^4+^ increases from +4 to +6 using its vacant d-orbitals to accept oxygen lone pairs forming Ti-O bonds. These six-fold structural units reach a high pressure and become the octahedra that finally combine into precipitate crystals. During condensation reactions, corner and edge sharing occurs as the octahedra agglomerate^2^. As the agglomeration of particles proceeds in the aqueous solution, its acidity plays a role that is crucial to the hydrolysis of Ti(RO)_4_^[22,23^. Under high acidity, Ti-OHˉ groups receive H from the solution and turn into ${{\rm{T}}{\rm{i}}-{\rm{O}}{\rm{H}}}_{2}^{+}$ groups (protonated groups) that combine easily with OHˉ groups from other TiO_6_ octahedra. The hydrogen bond among the protonated nanocrystallines could be attributed to aggregation of TiO_2_. Ti-O-Ti oxygen bridge bonds are formed when water molecules are eliminated (dehydration)^24^. The TiO_6_ octahedra must have a high degree of protonation in the rutile phase to form a dense and favorable orientation. Therefore, hydrolysis and dehydration reactions will be effectively catalyzed under highly acidic concentrations^3^. Furthermore, there must be high temperatures for crystal growth to proceed. Formation of the anatase phase preferred weak acidic conditions because of the lower surface energy of anatase whereas rutile phase did not prefer these conditions^23^. After the dehydration reaction, poor protonation results in a face-sharing anatase phase (Fig. 2(a)) while sufficient protonation results in corner and edge sharing in rutile phase (Fig. 2(b))^22^. The chemical reactions of hydrolysis and dehydration (condensation) in the hydrothermal synthesis process can be written as follows^23,24^.

1.1 $${\rm{Hydrolysis}}:{{\rm{Ti}}({\rm{OR}})}_{4}+4{{\rm{H}}}_{2}{\rm{O}}\to {{\rm{Ti}}({\rm{OH}})}_{4}+4{\rm{ROH}}$$

1.2 $${\rm{D}}{\rm{e}}{\rm{h}}{\rm{y}}{\rm{d}}{\rm{r}}{\rm{a}}{\rm{t}}{\rm{i}}{\rm{o}}{\rm{n}}:{{\rm{T}}{\rm{i}}({\rm{O}}{\rm{H}})}_{4}+{{\rm{T}}{\rm{i}}({\rm{O}}{\rm{H}})}_{4}\to {2{\rm{T}}{\rm{i}}{\rm{O}}}_{2}+4{{\rm{H}}}_{2}{\rm{O}}$$

1.3 $${{\rm{Ti}}({\rm{OH}})}_{4}+{{\rm{Ti}}({\rm{OR}})}_{4}\to 2{{\rm{TiO}}}_{2}+4{\rm{ROH}}$$

The entire reaction via the hydrothermal process between TBO precursor and water is^22,23^

1.4 $${{\rm{T}}{\rm{i}}({\rm{O}}{\rm{R}})}_{4}+2{{\rm{H}}}_{2}{\rm{O}}\to {{\rm{T}}{\rm{i}}{\rm{O}}}_{2}+4{\rm{R}}{\rm{O}}{\rm{H}},$$

where R is the component of a Ti (IV)-butoxide that designates a butyl-group with the general formula C_4_H_9_. Basically, optimum concentrations of acidity (OHˉ/H^+^ ratio) are crucial for crystal orientation. Recently, a similar approach with a hydrothermal solution using TBO, HCl and DI water, reported that most of the oxygen in TiO_2_ came from OH and OH_2_ groups, as confirmed by XPS data. The surface hydroxide ions contributed to the growth of 1D single crystalline TiO_2_ nanorods on an FTO substrate^25^. Another report indicated that rutile and anatase phases were the result of nanorod synthesis on a seed layer using a hydrothermal method with 1–2 pH and 4–5 pH synthesis solutions, respectively^18^.

### Experimental details

#### Preparation of seed layers

FTO substrates were ultrasonically cleaned sequentially for 15 min each in a 2% solution of alconox cleaning detergent and distilled water then acetone and finally isopropanol. Next, a flow of nitrogen gas passed over the FTO substrates and then an ultraviolet-ozone cleaning process was used for 30 min. The specific seed layers were coated on the cleaned FTO substrates by spin coating 0.15 M of titanium diisopropoxide bis(acetylacetonate) (TDB) in 1-butanal solvent at 3000 rpm for 30 s. The coated substrates were annealed at 125°C for 5 min. After that, the films were deposited by another spin coating of a 0.30 M TDB solution and annealed at 125°C for 5 min, this second spin coating process was repeated. Finally, the seeded substrates were then heated at 500°C for 30 min.

### Synthesis of TiO_2_ NRs

The hydrothermal process was carried out in Teflon liners (50 ml volume) contained in stainless steel autoclaves. In order to decrease the amount of toxic chemicals used, deionized (DI) water and 37% by weight hydrochloric acid (HCl) were mixed together at volume ratios of 2:1 and stirred for 5 min. Then, two titanium (IV) butoxide (TBO) precursors were prepared (0.7% and 1% per solution volume) and each was added to the hydrochloric acid mixture and stirred for another 5 min. Next, the FTO substrates with TiO_2_ seed layers were placed in Teflon liners. The autoclaves were heated at 150°C for 1, 1.5, 2 and 4 h and then water is circulated through the autoclave chamber jacket to cool down the chamber and its contents. Finally, the FTO films were removed from the Teflon liners, rinsed with distilled water, and dried under ambient conditions. Finally, various synthesis conditions were characterized by field emission scanning electron microscopy (FE-SEM), X-ray diffraction (XRD), and transmission electron microscopy (TEM) to investigate the morphology and crystalline structure of the prepared TiO_2_ NRs.

## Results and discussion

### Characterization of morphology by field emission scanning electron microscopy (FE-SEM)

**Figure 3:**
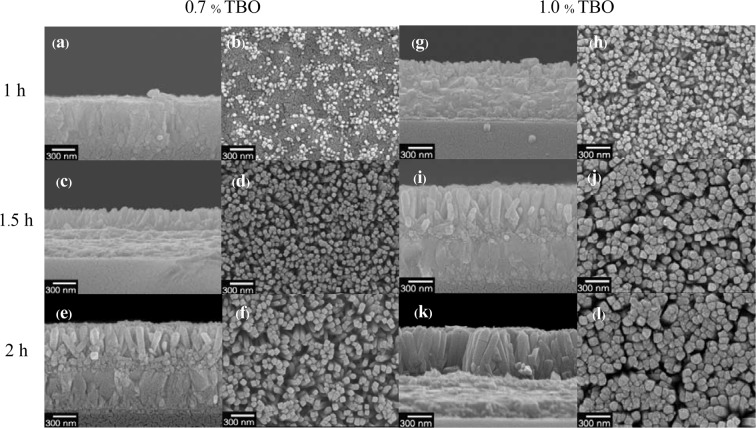
Cross-section and top views of FE-SEM images showing TiO_2_ nanorods obtained by hydrothermal reaction with 0.7% TBO heated for (**a**,**b**)1 h, (**c**,**d**) 1.5 h and (**e**,**f**) 2 h and with 1.0% TBO heated for (**g**,**h**)1 h, (**i**,**j**) 1.5 h and (**k**,**l**) 2 h.

FE-SEM images of the TiO_2_ NRs synthesized at different TBO concentrations and different heating times demonstrate the same trend. This trend, as shown in Fig. 3, is that the dimensions of the NRs increased as reaction time increased. The 1.0% TBO has a greater concentration of TBO molecules, which consequently provides a higher growth rate and initial nucleation than that of 0.7% TBO. This results in larger nanorod dimensions for the 1.0% TBO. The average diameters and lengths for 0.7% and 1.0% TBO NRs are illustrated in Table 1.

**Table 1:** Average diameter and length of TiO_2_ NRs prepared using a hydrothermal process at different TBO concentrations and heating times.

Concentration TBO TiO_2_ (%)	Hydrothermal reaction time (h)	Diameter (nm)	Length (nm)
0.7	1	39 ± 4	56 ± 11
0.7	1.5	51 ± 9	270 ± 20
0.7	2	66 ± 7	441 ± 30
1.0	1	49 ± 6	176 ± 17
1.0	1.5	78 ± 20	522 ± 58
1.0	2	96 ± 17	614 ± 27

**Figure 4:**
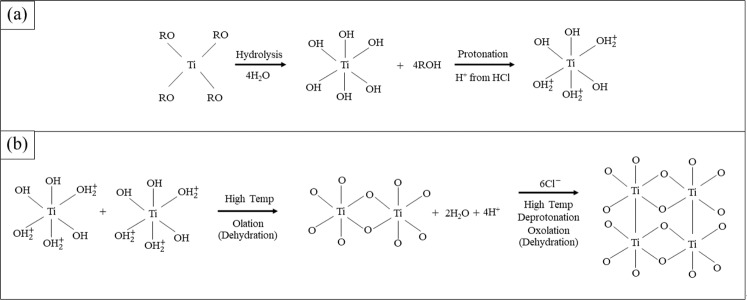
The role of HCl in the reaction mechanism of rutile growth during (**a**) hydrolysis and protonation and (**b**) olation and oxolation. R is the component of Ti (IV)-butoxide named butyl-group (Ti(OCH_2_CH_2_CH_2_CH_3_)_4_, [Ti(RO)_4_]) with the general formula C_4_H_9_.

At 1.5 h of hydrothermal reaction time, the length of the NRs extended significantly and grew moderately after that. On the other hand, the diameter showed little growth compared to the length. Let us consider the growth mechanism of NRs, which is a reaction that consists of 4 parts as shown in Fig. 4. The first part is hydrolysis, where groups bonded to Ti atoms are exchanged. Specifically, butyl groups [R] are replaced by hydroxyl groups [OH^−^]. The second part involves some Ti-OH groups receiving protons (H^+^) from HCl and becoming ${{\rm{T}}{\rm{i}}-{\rm{O}}{\rm{H}}}_{2}^{+}$ groups (protonated groups) that combine easily with the OHˉ groups of other TiO_6_ octahedra as we see in Fig. 4(a). This process is called protonation, which then proceeds to olation. This third step, olation, is dehydration or condensation, which combines compounds to form metal oxides and release water molecules. Olation usually occurs in highly acidic mediums thus it is attributed to producing long chains of highly protonated Ti-complexes. Finally, the formation of larger NRs as shown in Fig. 4(b) is called oxolation, which is another kind of condensation process. Oxolation contributes to the development of a lateral arrangement in less protonated solutions. During the initial reaction, the concentration of HCl is high in the earliest stage and therefore causes high protonation, which also leads to fast olation. The fact that olation takes place promptly results in long chains of TiO_6_ octrahedra with high positive charges. High amounts of positive charge would obstruct further olation because the chains are not stable. Therefore, Clˉ ions are a necessary contributor to the deprotonation of the cation due to their high electronegativity, which strongly attracts protons. During deprotonation, Clˉ ions trap the migrating protons thus lowering an increasingly positive charge caused by the condensation. More reactions occur that trigger the removal of OHˉ and result in H_2_O molecules. As a result of deprotonation, TiO_6_ octahedra form in a radial direction^22^. From SEM results in Fig. 3, noticeable growth in the length of the NRs can be observed between 1 h and 1.5 h. This increase indicates that olation was the dominant process between 1 h to 1.5 h and then after 1.5 h, olation slowed down. With an excess of protons in the long chains, the occurrence of oxolation could increase the diameter of the NRs and allow olation to continue lengthening the NRs. Based on our results, olation was found to be dominant in this hydrothermal process.

### Characterization of crystallinity by X-ray diffraction (XRD)

**Figure 5:**
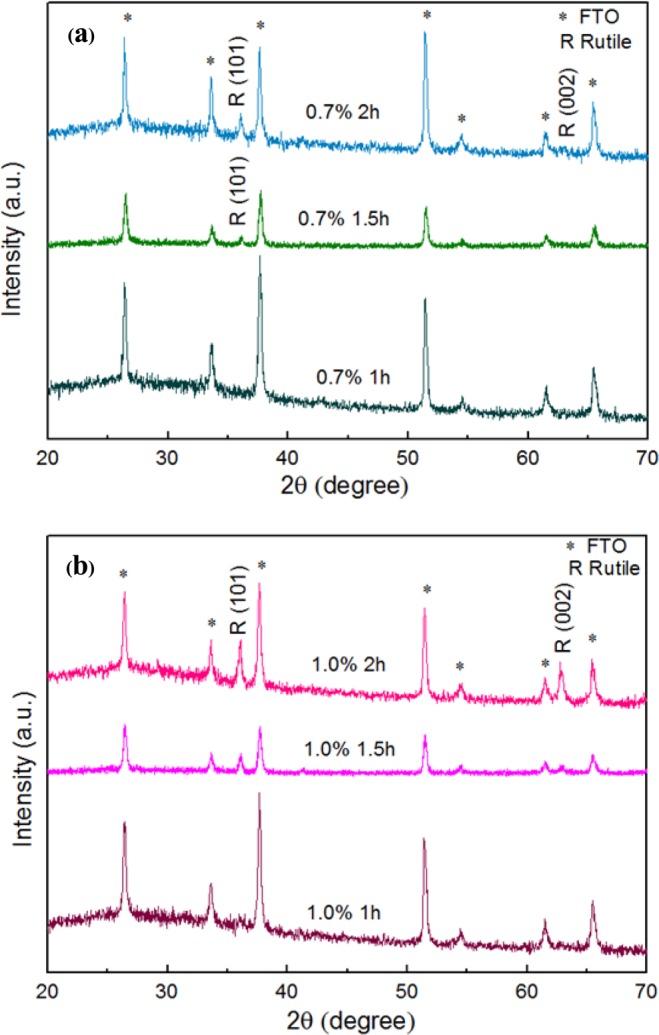
XRD pattern of (**a**) 0.7% TBO TiO_2_ NRs and (**b**) 1.0% TBO TiO_2_ NRs with reaction time 1 h, 1.5 h and 2 h, respectively.

The results show that the characteristic plane of (101) for tetragonal rutile structure emerges when the hydrothermal process with 0.7% TBO TiO_2_ NRs was conducted for 1.5 h. Furthermore, as time increased to 2 h, the minimal (002) plane appeared as shown in Fig. 5(a). Even though the seed layer is anatase^26^, NRs showed a rutile structure due to the pH of the solution. For the 1.0% TBO TiO_2_ NRs, Fig. 5(b) shows a (101) plane of rutile and a small (002) rutile peak when the duration of the reaction reached 1.5 h.

**Figure 6:**
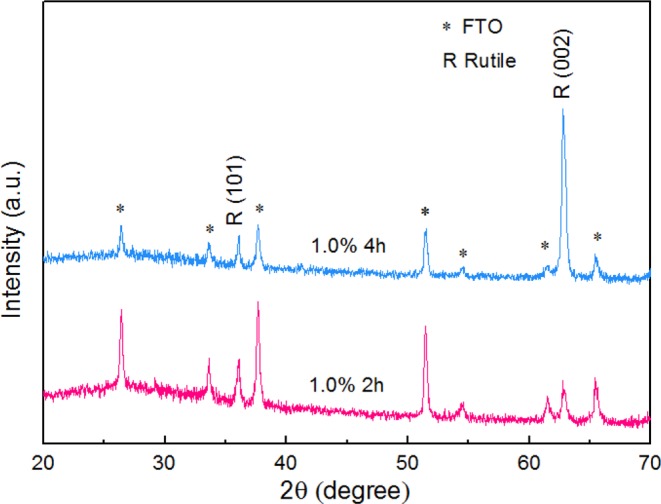
XRD pattern of 0.7% TBO TiO_2_ NRs with reaction time 2 h and 4 h.

When the hydrothermal process was prolonged to 2 h for 1.0% TBO TiO_2_ NRs, the intensity of the (002) peak was higher than at 1.5 h as shown in Fig. 5(b). Moreover, it was obvious that the (101) plane emerges before the (002) plane because (101) it is one of the lowest energy planes and can be more easily initiated. When the hydrothermal reaction was conducted for 4 h, the (002) intensity dramatically increased which demonstrates that the dominant plane of NRs on the seed layers was (002) rutile corresponding to previous results as shown in Fig. 6. The 0.7% TBO and the 1.0% TBO show high vertical alignment and homogeneous distribution of NRs, therefore they are well suited for observing optical properties. Generally, rutile structure shows a direct optical band gap and from the Ultraviolet–visible spectroscopy (UV-VIS) spectra we determine direct band gaps of 3.14 and 3.22 for 0.7% TBO NRs and 1.0% TBO NRs, respectively as shown in our previous report. These band gap values are characteristic of wide band gap semiconductor materials^17^. In addition, earlier research demonstrated that the significant transmittance for 0.7% TBO NRs was higher than for 1.0% which could well make them suitable for ETLs in PSCs^17^.

### Structural characterization by transmission electron microscopy (TEM)

**Figure 7:**
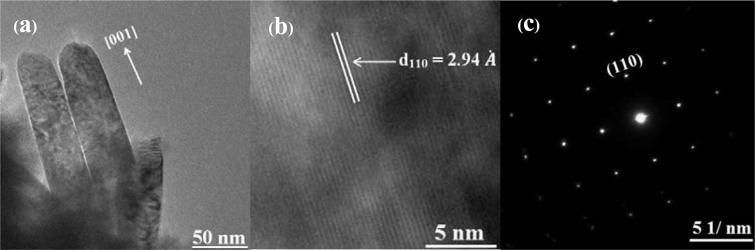
Image of 0.7% TBO TiO_2_ NRs hydrothermal for 1.5 h (**a**) TEM (**b**) HRTEM and (**c**) SAED pattern showing single crystalline rutile TiO_2_ in (**a**) and (**b**).

**Figure 8:**
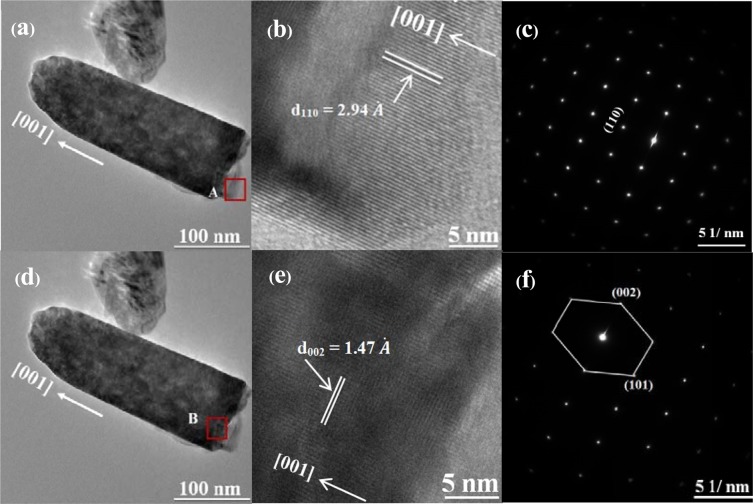
Image of 1.0% TBO TiO_2_ NRs (hydrothermal growth) for 1.5 h (**a**,**d**), TEM (**b**,**e**) HRTEM of “A” and “B” areas and (**c**,**f**) SAED of “A” and “B” areas.

Selected area electron diffraction (SAED) patterns in Figs. 7(c) and 8(e,f) show that 0.7% TBO NRs and 1.0% TBO NRs have single crystalline structures while the (002) plane is dominant for the large NRs with high crystallinity (see Fig. 6). The single-crystal architecture is significant for application in solar cells because the single crystal offers a fast pathway via the length of the NR with less grain boundary. In addition, fewer defect traps are present in single-crystal NRs compared to NRs with poor crystallinity which would lead to lower recombination^27^. Figure 7(a,b) presents high resolution transmission electron microscopy (HRTEM) results for 0.7% TBO NRs consisting of (110) growth along the [001] direction with d-spacing of 2.94 Å. This is also confirmed in Fig. 7(c) by SAED patterns for 0.7% TBO NRs, which is evidence of (110) plane orientation within the NRs. For 1.0% TBO NRs, Fig. 8(b) displays the orientation of the (110) face of the NRs observed at area “A” of Fig. 8(a). From investigation of the “B” area of 1.0% TBO NRs, shown in Fig. 8(d), we highlight the lattice fringes of (101) and (002) facets which were found and present these in Fig. 8(f). The (101) plane shows interplanar spacing of 2.24 Å while the (002) face exhibits a much lower d-spacing of 1.47 Å$.$ This is the reason why (002) is the prominent plane. This corresponded to XRD results showing that (101) and the (002) rutile peak could be detected in 1.0% TBO NRs after 1.5 h of hydrothermal treatment (Fig. 6).

**Figure 9:**
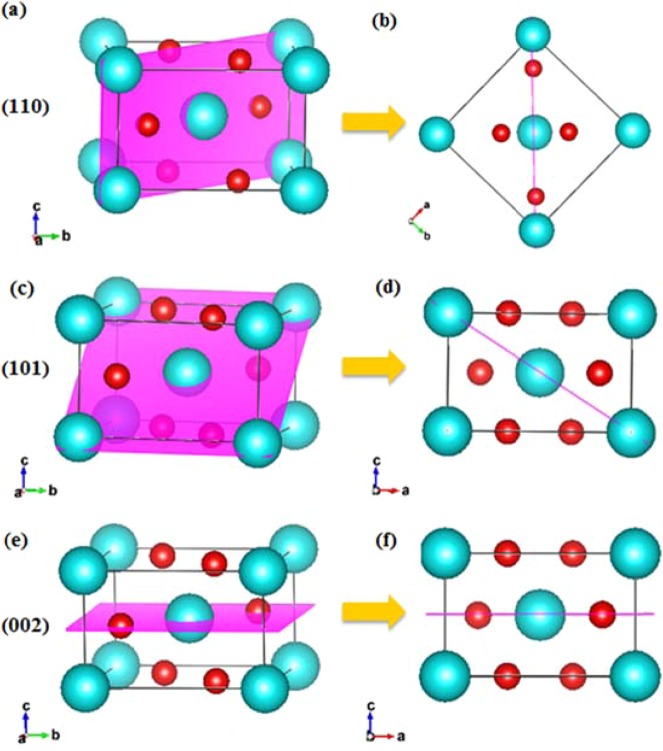
Dominant planes of rutile TiO_2_ structure (**a**) (110) plane in 3D space, (**b**) (110) plane in [001] direction, (**c**) (101) plane in 3D space, (**d**) (101) plane in [010] direction, (**e**) (002) plane in 3D space and (**f**) (002) plane in [010] direction.

**Figure 10:**
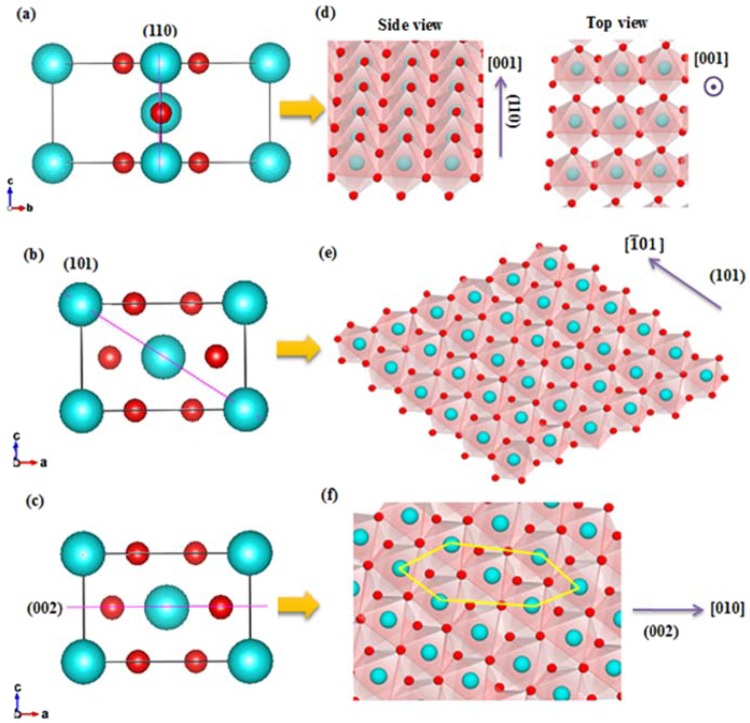
Dominant planes of rutile TiO_2_ structure (**a**) (110) plane in [$1\bar{1}0$] direction, (**b**) orientation of (101) plane, (**c**) (002) plane in [010] direction, (**d**) orientation of (110) plane, (**e**) (101) plane in [010] direction and (**f**) orientation of (002) plane.

In order to investigate the fundamental understanding behind the growth mechanism, an explanation of related planes in rutile structure will be considered. Dominant planes of the tetragonal rutile TiO_2_ structure in this work consist of (110), (101), and (002). Atomic arrangements of these faces in three dimensions and side views are shown in Fig. 9. For clear illustration of the plane orientation in the crystal structures, alignment of TiO_6_ octahedra into planes and growth direction of each facet are shown in Fig. 10. It can be seen that the (110) orientation in Fig. 10(a) corresponds to the (110) bright spot in Fig. 7(c). Figure 8(c) represents lattice fringes that correspond to faces (101) and (002) in Fig. 8(f).

One aspect to consider when describing crystal growth phenomena is the surface energy, which is a relative measurement. The definition of surface energy is the surface excess free energy per unit area of a crystal facet. It determines the equilibrium shape of crystals and crystal development^28^. Generally, internal attraction between bulks is balanced but at the interfacial area attraction energy is unstable. Therefore, facial atoms form themselves into films at the surface resisting external force. Further crystallization to form a new surface requires surface energy in order to break the intermolecular force of the surface^29^. The energy depends on the number of atoms at the surface, thus surface energy is dependent on direction. Recently, several approaches have been performed to clarify surface energy of rutile TiO_2_. On the basis of previous reports, (110) rutile was found to be the lowest surface energy face with the most stability^30^. In this work, (110) could not be detected by XRD but could be observed using the SAED mode of TEM because (110) orients in a direction that does not promote constructive interference of X-ray beams. This results in diffraction in unsuitable directions, which cannot be detected by XRD.

According to XRD and TEM results, it is obvious that the (101) plane occurred first followed by the formation of the (002) plane when the hydrothermal reaction time had increased. This effect can be explained by geometrical configurations of (101) and (002) surfaces. The (101) surface possessed fivefold coordinated titanium atoms (pentacoordinated; Ti(5)) while the (002) facet was composed of fourfold coordinated titanium atoms (Ti(4)), therefore the (002) plane required more energy to continue the growth into TiO_6_ octahedra relative to (101). In other words, the (002) face had a higher energy surface than the (101) face^30,31^. Moreover, there are more incomplete Ti(5) atoms on the (101) plane than on (110), therefore the (110) surface demanded less energy and fewer Ti-complex molecules to bond and complete its development into a new plane. On the other hand, the arrangement of atoms on the (110) plane is complete with fourfold coordinated Ti atom and twofold coordinated oxygen atoms. For these reasons, the surface energy of (101) (1.85 J/m^2^) is greater than that of (110) (1.78 J/m^2^) and the (110) facet is the most stable surface^30,32^. When the hydrothermal process proceeded for 4 h, (002) became intense. While (101) stopped growing, as supported by XRD data in Fig. 6. Geometric reasons can be used to reveal the mechanism of these phenomena. Because (101) orients against [001] direction with an angle as shown in Figs. 9(c) and 10(b,e), development of (101) results in increases to the NRs in terms of width and length but the specific area is restricted from growing laterally. When the diameter of the NRs grew and their walls contacted with the neighboring NRs, the (101) plane could no longer extend.

Moreover, an enhanced supply of TiO_6_ octahedra on the surface could restrict further oxolation of [TiO(OH_2_)_5_]^2+^ complex on the (101) surface^25^. On the other hand, (002) with small d-spacing aligns perpendicular to the [001] direction (Fig. 9(f)) and the preferential growth of rutile NRs is in the [001] direction. The number of (002) planes increases with the increasing length of the NRs and is not restricted by the lateral area. Therefore, the extreme growth of NRs in the [001] direction results in a greater number of (002) faces. This is the reason why the (002) plane is dominant and corresponds to XRD data from Fig. 6 which shows the high intensity of the (002) peak for NRs after a hydrothermal reaction conducted for 4 h. Another approach suggests that HCl plays a role in reducing the surface energy of the crystal plane side wall and supporting anisotropic growth in the [001] direction^33^.

Our results of the growing well-aligned TiO_2_ single-crystal NRs with optimized sizes and spaces open up a promising approach to achieve PSCs with high PCE. The obtained TiO_2_ is capable of being a promising alternative to be employed as an efficient ETL. One device of the TiO_2_ NRs-based PSCs being developed was determined to achieve a PCE of 11.39%. Further details of the effective TiO_2_-based PSCs are evaluating and a paper is being prepared.

## Conclusions

Dimensions of NRs increased as reaction time and TBO concentration increased. The TEM and SEM results were able to support XRD results and describe the growth mechanisms during hydrothermal reaction. At 1.5 h of hydrothermal reaction time, the length of the NRs was seen to have extended significantly and grew moderately after that. This significant growth in length was noted between 1 h and 1.5 h indicating that olation (the formation of long chains of highly protonated Ti-complexes) occurred mostly during this time period then slowed down at the 2 h mark. Oxolation, leading to the construction of a lateral arrangement of [TiO(OH2)5]^2+^ complexes, could assist olation to continue which means NR diameters would be forced to expand as length increases. With an excess of protons in the long chains, the occurrence of oxolation could increase the diameter of the NRs and allow olation to continue lengthening the NRs. However, an enhanced supply of TiO_6_ octahedra on the surface could restrict further oxolation on the surface. Due to oxolation taking place less than olation, the diameters of the NRs were smaller than the length. The (110) plane growing parallel to the length of the NRs is a result of olation of (002) while (101) aligning in a lateral direction is a result of oxolation. Selected area electron diffraction (SAED) patterns illustrate that the 0.7% TBO NRs and 1.0% TBO NRs have single crystalline structure growth along [001] while the (002) plane is the dominant facet. The single-crystal architecture for 0.7% TBO NRs obtained after 1.5 h of hydrothermal growth is significant for application in solar cells due to the high transmittance of visible light and the fact that the structure offers a fast electron pathway via the length of the NRs with less grain boundary. In addition, fewer defect traps are present in single-crystal NRs compared to NRs with poor crystallinity, which would lead to lower recombination.
